# Pre-pregnancy BMI, gestational weight gain and body image are associated with dietary under-reporting in pregnant Japanese women

**DOI:** 10.1017/jns.2018.3

**Published:** 2018-04-02

**Authors:** Mie Shiraishi, Megumi Haruna, Masayo Matsuzaki, Ryoko Murayama, Satoshi Sasaki

**Affiliations:** 1 Children and Women's Health, Osaka University, Osaka, Japan; 2 Midwifery and Women's Health, The University of Tokyo, Tokyo, Japan; 3 Advanced Nursing Technology, The University of Tokyo, Tokyo, Japan; 4 Social and Preventive Epidemiology, The University of Tokyo, Tokyo, Japan

**Keywords:** BMI, Dietary under-reporting, Gestational weight gain, Pregnancy, Dietary surveys, Nutritional epidemiology, DHQ, Diet History Questionnaire, FHLC, Fetal Health Locus of Control Scale, GSES, General Self-Efficacy Scale

## Abstract

Dietary under-reporting is a common problem when using self-reported dietary assessment tools. However, there are few studies regarding under-reporting during pregnancy. This study aimed to explore the demographic and psychosocial characteristics related to dietary under-reporting in pregnant Japanese women. A cross-sectional study was conducted between 2010 and 2011 at a university hospital in Tokyo, Japan. Nutrient intake was assessed using a self-administered Diet History Questionnaire (DHQ), which had questions about the consumption frequency and portion size of selected food items. The 24-h urinary excretion levels of urea N and K were used as the dietary protein and K intake reference values, respectively. Under-reporting of protein and K was defined as the bottom 25 % of the reporting accuracy (the ratio of reported intake on the DHQ to the estimated intake based on urinary excretion). Under-reporters were defined as participants who under-reported both protein and K intake. Multiple logistic regression analysis was performed to examine the factors associated with under-reporters. Of 271 healthy women at 19–23 weeks of gestation, thirty-five participants (12·9 %) were identified as under-reporters. Under-reporters had a lower pre-pregnancy BMI (adjusted OR (AOR) = 0·81) and lower gestational weight gain (AOR = 0·82); they also reported managing their gestational weight gain with the aim to return to their pre-pregnancy weight soon after childbirth (AOR = 2·99). Healthcare professionals should consider the potential for dietary under-reporting and the possible related factors when assessing the dietary intakes of pregnant Japanese women using self-administered questionnaires.

Self-reported dietary assessment tools have systematic measurement errors. A common measurement error is under-reporting, which occurs especially in women^(^^1^^,^^2^^)^. Dietary under-reporting is a significant problem that could lead to erroneous conclusions regarding diet–disease associations^(^^3^^)^. In the clinical setting, dietary under-reporting may lead to incorrect assessment and instructions by healthcare professionals. Since appropriate nutrition during the prenatal period is important for fetal development and prevention of pregnancy complications, provision of incorrect nutrition-related instruction can negatively affect fetal and maternal health. Three studies on pregnant women, conducted in Indonesia, Ireland and USA, indicated that 14–44 % of the women were dietary under-reporters, based on the ratio of energy intake to the estimated BMR^(^^4^^–^^6^^)^. In non-pregnant women, the prevalence of dietary under-reporting via FFQ ranges from 12 to 54 %^(^^7^^–^^16^^)^. In Japan, 18–32 % of non-pregnant women have been found to under-report dietary intake^(^^13^^–^^16^^)^.

Since the reporting accuracy of dietary intake varies according to the demographic and psychosocial characteristics of the respondents, researchers need to understand the potential risk and the related factors of under-reporting in their specific target population. The following factors related to under-reporting have been identified in non-pregnant populations: demographic factors (sex and age)^(^^1^^)^, socio-economic factors (household income and educational level)^(^^17^^–^^19^^)^, dietary restrictions (previous and current)^(^^8^^)^, weight-related factors (BMI and body image)^(^^1^^,^^13^^,^^16^^,^^20^^,^^21^^)^ and psychosocial factors (social desirability, social approval motivation and self-efficacy)^(^^2^^,^^11^^,^^22^^,^^23^^)^. Previous studies on pregnant women showed that high BMI and low education levels were associated with dietary under-reporting^(^^4^^–^^6^^)^; however, a detailed examination including behavioural and psychological factors has not been conducted in this population. Further, these related factors vary depending on the target population or country; some factors related to under-reporting in pregnant women may not exist in non-pregnant populations.

The attitudes of pregnant women regarding diet for themselves and their babies may be easily changed. Their attitudes toward diet are often affected by particular factors associated with pregnancy, such as pregnancy-associated nausea, weight management and beliefs pertaining to the health of their babies^(^^24^^,^^25^^)^. In Japan, many pregnant women are careful not to gain excessive weight during pregnancy^(^^25^^)^ because they know that it may lead to pregnancy complications, difficult deliveries and postpartum weight retention. However, such attitudes toward weight gain during pregnancy might also influence dietary reporting accuracy, as has been reported regarding attitudes toward weight reduction in non-pregnant populations^(^^1^^)^. In addition, a strong belief that maternal health behaviour has a positive effect on the well-being of babies might influence the reporting accuracies due to socially desirable responses. Identifying the factors related to under-reporting during pregnancy is important in yielding reliable data for dietary research because non-random dietary under-reporting could distort diet–disease associations.

The self-administered Diet History Questionnaire (DHQ) is the only dietary assessment tool that has been validated among pregnant Japanese women^(^^26^^)^. In order to use the DHQ appropriately, it is necessary to gain a better understanding of the reporting accuracy and factors related to under-reporting during pregnancy.

For identification of under-reporters, energy expenditure is commonly measured as a reference value of the energy intake estimated from dietary assessment tools. The doubly-labelled water method is a ‘gold standard’ method used to measure energy expenditure^(^^27^^)^, and the BMR, which is calculated from the individual's weight, height and age, is often used to evaluate energy expenditure. However, these methods cannot be used in pregnant women due to unverified safety and the difficulty of estimating energy expenditure based on weight^(^^28^^)^. As a reference for evaluating the reporting accuracy of energy intake, the reporting accuracies of protein and K intakes are often evaluated using 24-h urine collection^(^^29^^,^^30^^)^. This method has two advantages: protein and K can reflect a wide variety of foods, and 24-h urine collection is a method with comparatively few burdens compared with the doubly-labelled water method. Thus, measurement of 24-h urine markers of protein (urea N) and K is an appropriate method to identify dietary under-reporting during pregnancy.

The objective of the present study was to examine the factors related to dietary under-reporting in the DHQ among pregnant Japanese women, using 24-h urine markers.

## Methods

### Overview of the recruitment process and study design

A cross-sectional study was conducted at a university hospital in Tokyo, Japan, between June 2010 and June 2011. Healthy Japanese women with singleton pregnancies, who were aged 20 years or older, and who had adequate Japanese literacy skills, were recruited at 15–19 weeks of gestation. Women who provided written informed consent participated in the investigation at 19–23 weeks of gestation. In Japan, nutritional guidance for increased nutrient needs during pregnancy is often performed around 20 weeks of gestation after nausea and vomiting are relieved. Because conducting appropriate nutritional assessment at this period is important for ensuring appropriate individual dietary instructions, we set 19–23 weeks of gestation as the target period of this investigation. The reason for recruitment at 15–19 weeks of gestation was to allow time for giving detailed instructions on the method of urine collection and provide all necessities before the investigation. The exclusion criteria included women with diseases that affected dietary intake, such as hyperemesis, diabetes and hypertension. In addition, women with psychiatric disorders such as depression and schizophrenia were excluded.

Upon recruitment (15–19 weeks of gestation), the participants received written and verbal instructions on the method of urine collection and the necessity of obtaining a complete 24-h urine collection. The participants were provided with 3 litre, 1 litre and 50 ml plastic bottles, as well as 350 ml cups and a dropper. A 24-h urine collection was conducted on the preceding day or within 5 d before the next pregnancy check-up (19–23 weeks of gestation). On the day before a urine collection, the participants received a reminder call to explain the urine collection procedure again. Participants were asked to discard their first urine specimen on the morning of collection and to collect all specimens for the next 24 h. After all urine was collected, the urine volume was marked as a horizontal line on the 3 litre bottle with a felt-tipped marker. After the well-stoppered 3 litre bottle was shaken up and down at least ten times, a sample of the pooled urine was transferred to the 50 ml plastic bottle using a dropper. These procedures were performed by the participants. A researcher received each 50 ml plastic bottle with urine sample as well as the empty marked 3 litre bottle.

Each participant completed the questionnaire while waiting for an antenatal check-up at 19–23 weeks of gestation. Participants who did not have sufficient time to answer the questionnaires completed them after returning home and submitted them by mail within 7 d. We resolved missing and unclear data directly when the questionnaires were submitted or by telephone interview. We reviewed the participants’ medical charts to obtain information regarding their pregnancies.

This study was conducted according to the guidelines laid down in the Declaration of Helsinki and all procedures involving human subjects/patients were approved by the ethics committee of the university (no. 3197). Each participant received detailed information about the study protocol, and then provided written informed consent.

### Dietary assessment

The DHQ was used for the dietary assessment of pregnant Japanese women in this study. The DHQ was designed to assess dietary intake over the previous month in the Japanese adult population^(^^31^^–^^34^^)^ and has been previously validated in pregnant Japanese women^(^^26^^)^. The DHQ-measured unadjusted protein and K intake levels in the second trimester were significantly correlated with the corresponding 24-h urinary levels (*r*_s_ 0·307 and *r*_s_ 0·342, respectively)^(^^26^^)^. The DHQ is a twenty-two-page structured questionnaire that measures the daily intakes of 150 foods and selected nutrients. Items were derived from primary data of the National Nutrition Survey of Japan and various Japanese recipe books for Japanese dishes^(^^31^^)^. Protein intake was calculated mainly from cereals, nuts and pulses, fish and shellfish, meat and eggs. K intake was calculated mainly from cereals, potatoes, nuts and pulses, vegetables, fruits, fish and shellfish, meat and eggs. Eight eating frequency responses were listed, ranging from ‘more than twice per d’ to ‘almost never’. The following five portion size responses were listed: less than half of the standard portion size, 0·7–0·8 times the standard portion size, standard portion size, 1·2–1·3 times the standard portion size, and more than 1·5 times the standard portion size. The standard portion sizes were derived from several recipe books for Japanese dishes. For instance, the standard portion size of milk is shown as a cup of 150 ml. The DHQ also includes questions regarding general dietary behaviours such as seasoning preferences, and usual cooking methods for fish and shellfish, meat, eggs and vegetables. This information regarding general dietary behaviours was used for estimation of dietary intake of five seasonings such as sugar and soya sauce. Estimates of nutrient intakes were calculated from the consumption frequencies and portion sizes of the foods listed using an *ad hoc* computer algorithm developed for the DHQ^(^^31^^–^^34^^)^.

Pregnant women in their second trimester are recommended to have an additional energy intake of 1050 kJ/d (250 kcal/d) compared with non-pregnant women. Therefore, the recommended energy intakes are 7950–9200 kJ/d (1900–2200 kcal/d) for 20- to 29-year-old women and 8370–9410 kJ/d (2000–2250 kcal/d) for 30- to 49-year-old women^(^^35^^)^. The values vary by the individual physical activity level. We excluded participants who reported an extremely unrealistic energy intake, such as small eaters due to severe nausea and vomiting during pregnancy; that is, we excluded women in whom the reported energy intake was less than half the energy requirement for the lowest physical activity category (3970 or 4180 kJ/d; 950 or 1000 kcal/d) or more than 1·5 times the energy requirement for moderate physical activity (13810 or 14120 kJ/d; 3300 or 3375 kcal/d) according to the ‘Dietary Reference Intakes for Japanese’^(^^35^^,^^36^^)^.

### Biological measurements

A single 24-h urine collection was conducted for measurements of urea N and K levels. The urine samples were stored at −80°C within 8 h of collection, until the analyses. Urinary urea N levels were measured by the urease-LEDH method using an Iatro-LQ UN (A) II instrument (Mitsubishi Kagaku Bio-Chemical Laboratories, Inc.). The urea N levels in the 24-h urine sample were used to estimate the amount of dietary protein. Urinary K levels were measured by ion-selective electrodes. These assays were conducted using an automated analyser (BM6050; JEOL) from Mitsubishi Kagaku Bio-Chemical Laboratories, Inc.

We excluded women who did not meet the following criterion for acceptable 24-h urine collection: 10·8–25·2 mg/kg of creatinine excretion divided by body weight^(^^37^^)^. Urinary creatinine excretion was measured by the enzyme method using an Iatro-LQ CRE (A) II instrument (Mitsubishi Kagaku Bio-Chemical Laboratories, Inc.).

### Anthropometric measurement

The participants’ weight was measured using a DC320 weight scale (Tanita Corp.) at the 19- to 23-week antenatal check-up. In this study, weight change during pregnancy refers to the difference between the weight at the check-up at 19–23 weeks of gestation and the pre-pregnancy weight ((weight at the check-up at 19–23 weeks of gestation) – (pre-pregnancy weight)).

### General questionnaires

We obtained the following demographic and lifestyle information using a self-administered questionnaire: age, gestational age, educational level, height, pre-pregnancy weight, smoking habits during pregnancy, pregnancy-associated nausea and vomiting, dietary restrictions, habit of skipping meals, habit of making own meals, and regular supplement usage. Pre-pregnancy BMI was calculated from the self-reported pre-pregnancy weight and height. The participants were classified as being underweight (BMI < 18·5 kg/m^2^), normal weight (18·5 ≤ BMI < 25·0 kg/m^2^) and overweight or obese (BMI ≥ 25·0 kg/m^2^) based on the WHO criteria. We defined regularly skipping meals as forgoing meals including a staple food, such as rice or bread, two or more times per week. Likewise, regularly skipping breakfast was defined as forgoing breakfast including a staple food, two or more times per week.

We asked questions regarding the participants’ attitudes toward gestational weight gain and whether they managed their weight gain to prevent pregnancy complications and difficult deliveries or to return to their pre-pregnancy weight soon after childbirth.

Questions regarding potential psychosocial factors related to dietary under-reporting included social desirability, social approval motivation, self-efficacy and the fetal health locus of control. Social desirability was assessed using the Japanese version of the Marlowe–Crowne Social Desirability Scale^(^^38^^)^. In the Japanese version, ten items were selected from the original thirty-three items, according to the Japanese cultural background^(^^38^^,^^39^^)^. We used five-point Likert scale responses ranging from ‘disagree strongly’ to ‘agree strongly’ (0–40 points), with higher scores indicating a higher level of social desirability. Cronbach's α in the present study was 0·715.

Social approval motivation was assessed using the Japanese version of the Revised Martin–Larsen Approval Motivation Scale^(^^40^^,^^41^^)^. This scale consists of twenty items, answered using five-point Likert scale responses ranging from ‘disagree strongly’ to ‘agree strongly’ (20–100 points). Higher scores indicate a greater need for social approval. Cronbach's α in the present study was 0·737.

Self-efficacy was assessed using the General Self-Efficacy Scale (GSES), the validity and reliability of which have been established^(^^42^^)^. This scale consists of sixteen true–false questions divided into three subscales: positive attitude to activities (seven items; e.g. ‘I tend to carry out my work with confidence’), anxiety about failure (five items; e.g. ‘I often feel depressed when I recall past mistakes and bad experiences’), and social position of abilities (four items; e.g. ‘I have better abilities than my friends’). Higher scores indicate a higher level of self-efficacy (0–16 points). Cronbach's α in the present study was 0·805.

Fetal health locus of control reflects pregnant women's locus of control beliefs toward the health of their babies; this was assessed using the Japanese version of the Fetal Health Locus of Control Scale (FHLC). The validity and reliability of the FHLC have already been established^(^^43^^,^^44^^)^. The Japanese version of the FHLC consists of fifteen items categorised into internal, chance, and supernatural subscales, each containing five items. The internal subscale indicates that the well-being of a woman's fetus is principally under her own behavioural control. The chance subscale indicates that the well-being of a woman's fetus is chiefly due to fate or chance. The supernatural subscale indicates that the well-being of a woman's fetus is primarily influenced by the divine. Scores are calculated for each component, and higher scores reflect stronger beliefs in that particular locus of control for determining fetal health. In this study, we used only the internal subscale of the FHLC. Items are rated using a six-point Likert scale ranging from ‘disagree strongly’ to ‘agree strongly’ (5–30 points). Cronbach's α in the present study was 0·884.

### Identification of dietary under-reporters

The reporting accuracy was calculated as the ratio of reported intake from the DHQ to the estimated intake based on the 24-h urinary excretion levels. Protein intake estimated from 24-h urinary excretion was calculated as (24-h urinary urea N × 7·72) g^(^^45^^)^ divided by 0·85, assuming that 85 % of the ingested protein is excreted in the urine at 23 weeks of gestation^(^^46^^)^. K intake estimated from urinary excretion was calculated as the 24-h urinary K level divided by 0·77, assuming that 77 % of the ingested K is excreted in the urine^(^^47^^)^. The K excretion rate for non-pregnant women was used, because the change associated with pregnancy is very small^(^^48^^)^. A reporting accuracy of <1·0 is theoretically considered as under-reporting, while accuracies of >1·0 and 1·0 are defined as over-reporting and accurate reporting, respectively^(^^49^^)^. In the present study, under-reporting of protein and K was defined as the bottom 25 % of the reporting accuracy based on a previous review^(^^3^^)^ that observed under-reporting in an average of 25 % of non-pregnant women and other previous studies ^(^^4^^–^^16^^)^. Herein, dietary under-reporters were defined as participants who under-reported both protein and K intake.

### Statistical analyses

Multiple logistic regression analysis was performed to examine the factors related to dietary under-reporters. Before performing this analysis, Student's *t* test, the Mann–Whitney *U* test, the χ^2^ test, and Fisher's exact test were conducted to identify potential variables related to under-reporters. Variables with *P* < 0·20 in the univariate analyses were selected as variables for the multiple logistic regression analysis. These variables were checked for multicollinearity. If the correlation coefficient of any two variables was greater than 0·50 or any two categorical variables were significantly correlated by the χ^2^ test, either variable was excluded from the model.

All statistical analyses were conducted using IBM SPSS Statistics for Windows, version 21.0 (IBM Japan). All statistical tests were two-sided and *P* values <0·05 were considered statistically significant.

## Results

A total of 398 pregnant women were recruited in the present study; 326 (81·9 %) provided written informed consent, answered the questionnaires and collected their urine. Of them, fifty-five were excluded from the analysis: thirty-one missed at least one urine collection, eight did not meet the creatinine criteria, seven had severely under-reported energy intakes, five dropped out, and four had missing data. Thus, data from 271 (68·1 %) healthy singleton pregnant women were analysed. No one met the criterion of dietary over-reporting.

The reporting accuracies of protein and K intakes were calculated as the ratios of reported dietary intake from the DHQ to the estimated dietary intake from 24-h urinary excretion levels (Table 1). The 25th percentiles of reporting accuracies were 0·77 for protein and 0·64 for K. A total of sixty-seven pregnant women were found to under-report protein intake (<0·77), and sixty-seven women were found to under-report K intake (<0·64). Of the pregnant women, thirty-five (12·9 %) under-reported both protein and K intakes, and were identified as dietary under-reporters. Among the dietary under-reporters, four (11·4 %) were pre-pregnancy underweight (BMI <18·5 kg/m^2^), thirty (85·7 %) were of normal weight (18·5 ≤ BMI < 25·0 kg/m^2^) and one (2·9 %) was overweight (BMI ≥ 25·0 kg/m^2^). No relationship between under-reporters and their pre-pregnancy BMI was found.

**Table 1.** Reporting accuracies* of protein and potassium intakes (*n* 271) (Mean values and standard deviations; medians and interquartile ranges)

	Mean	sd	Median	Interquartile range
Protein	0·98	0·30	0·92	0·77–1·15
K	0·89	0·36	0·82	0·64–1·06

* Reporting accuracy is the ratio of reported intake from the Diet History Questionnaire to the estimated intake based on 24-h urinary excretion levels.

Table 2 summarises the participant characteristics. The mean age was 34·5 (sd 3·9) years, and 65·3 % were primigravida. The rate of university graduates or above was 50·9 %. Of the participants, 29 % had pregnancy-associated nausea and vomiting. Women with nausea and vomiting or who were overweight or obese had significantly lower gestational weight gain at 19–23 weeks of gestation.

**Table 2.** Characteristics of participants (Mean values, standard deviations and ranges; numbers of participants and percentages)

	All participants (*n* 271)			Under-reporters (*n* 35)			Normal reporters (*n* 236)			
	Mean	sd	Range	Mean	sd	Range	Mean	sd	Range	P*
Age (years)	34·5	3·9	23–44	34·6	3·6	29–44	34·4	4·0	23–44	0·783
Gestational age (weeks)	20·3	1·1	19–23	20·5	1·3	19–23	20·3	1·1	19–23	0·376
Parity: primigravida										1·000
*n*	177			23			154			
%	65·3			65·7			65·3			
Education level										
High school/junior or technical college										0·250
*n*	133			14			119			
%	49·1			40·0			50·4			
University or above										
*n*	138			21			117			
%	50·9			60·0			49·6			
Working										0·202
*n*	120			12			108			
%	44·3			34·3			45·8			
Height (cm)	158·8	5·1	147–172	158·4	5·1	147–168	158·9	5·1	147–172	0·605
Pre-pregnancy BMI (kg/m^2^)	20·4	2·4	16·4–34·4	19·8	1·7	17·1–26·0	20·5	2·5	16·4–34·4	0·043
Underweight (BMI < 18·5 kg/m^2^)										0·284
*n*	52			4			48			
%	19·2			11·4			20·3			
Normal weight (18·5 ≤ BMI < 25·0 kg/m^2^)										
*n*	203			30			173			
%	74·9			85·7			73·3			
Overweight or obese (BMI ≥ 25·0 kg/m^2^)										
*n*	16			1			15			
%	5·9			2·9			6·4			
Weight change during pregnancy (kg)†	2·35	2·47	−8·7 to 10·4	1·69	2·41	−3·7 to 5·9	2·45	2·46	−8·7 to 10·4	0·087
Management of gestational weight gain with the aim to return to pre-pregnancy weight soon after childbirth										0·012
*n*	51			12			39			
%	18·8			34·3			16·5			
Smoking during pregnancy										1·000
*n*	1			0			1			
%	0·4			0·0			0·4			
Pregnancy-associated nausea or vomiting										0·265
*n*	79			13			66			
%	29·2			37·1			28·0			
Regularly skipping meals‡										0·193
*n*	112			18			94			
%	41·3			51·4			39·8			
Regularly skipping breakfast‡										0·065
*n*	87			16			71			
%	32·1			45·7			30·1			
Making own meal										1·000
*n*	246			32			214			
%	90·8			91·4			90·7			
Regular supplement usage										
Folic acid supplements										0·670
*n*	138			19			119			
%	50·9			54·3			50·4			
Multivitamin supplements										0·784
*n*	67			8			59			
%	24·7			22·9			25·0			
Fe supplements										0·808
*n*	65			9			56			
%	24·1			25·7			23·8			
MCSD (score)§	28·9	4·8	11–40	28·5	4·5	17–36	29·0	4·9	11–40	0·519
MLAM (score)ǁ	56·8	7·2	5–75	56·2	7·0	40–73	56·9	7·3	5–75	0·612
GSES (score)¶	8·7	3·7	0–16	7·7	3·3	2–15	8·8	3·7	0–16	0·080
FHLC, internal subscale (score)**	27·3	2·9	16–30	27·0	3·0	20–30	27·4	2·9	16–30	0·495

MCSD, Marlowe–Crowne Social Desirability Scale; MLAM, Revised Martin–Larsen Approval Motivation Scale; GSES, General Self-Efficacy Scale; FHLC, Fetal Health Locus of Control Scale.

* Student's *t* test, χ^2^ test or Fisher's exact test.

† Weight change during pregnancy refers to the difference between weight at the check-up at 19–23 weeks of gestation and the pre-pregnancy weight: ((weight at the check-up at 19–23 weeks of gestation) – (pre-pregnancy weight)).

‡ Regularly skipping meals (breakfast) was defined as forgoing meals (breakfast) including a staple food, such as rice or bread, two or more times per week.

§ Higher scores on the MCSD indicate a higher level of social desirability (0–40 points).

ǁ Higher scores on the MLAM indicate a greater need for social approval (20–100 points).

¶ Higher scores on the GSES indicate a higher level of self-efficacy (0–16 points).

** Higher scores on the FHLC reflect stronger beliefs in that particular locus of control for determining fetal health. The internal subscale indicates that the well-being of a woman's fetus is principally under her own behavioural control (5–30 points).

The median (interquartile range) levels of reported dietary intake and 24-h urinary excretion are shown in Table 3. The daily intakes of total energy, protein and K were significantly lower in the under-reporters than in the normal reporters (median intake, 5979 *v.* 7707 kJ/d (1429 *v.* 1842 kcal/d), 48·08 *v.* 64·30 g/d, and 1·66 *v.* 2·23 g/d, respectively). The protein and K intakes were significantly positively correlated with energy intake (*r* 0·856, *P* < 0·001 and *r* 0·783, *P* < 0·001, respectively). On the other hand, the urinary urea N and K levels were significantly higher in the under-reporters than in the normal reporters (median level, 7·46 *v.* 6·63 g/d, and 2·39 *v.* 1·78 g/d, respectively).

**Table 3.** Nutrient intakes estimated from the self-administered Diet History Questionnaire and 24-h urinary excretion levels (Medians and interquartile ranges)

	All participants (*n* 271)		Under-reporters (*n* 35)		Normal reporters (*n* 236)		
	Median	Interquartile range	Median	Interquartile range	Median	Interquartile range	P*
Nutrient intakes							
Energy							
kJ/d	7460	6431–8640	5979	5230–6456	7707	6581–8786	<0·001
kcal/d	1783	1537–2065	1429	1250–1543	1842	1573–2100	<0·001
Protein (g/d)	61·70	53·05–74·19	48·08	42·68–56·07	64·30	55·33–76·16	<0·001
K (g/d)	2·16	1·73–2·59	1·66	1·38–1·94	2·23	1·79–2·73	<0·001
Urinary markers							
24-h total urine volume (ml/d)	1320	1060–1690	1330	1060–1800	1315	1046–1690	0·701
Urea N (g/d)	6·77	5·66–7·90	7·46	6·60–8·78	6·63	5·52–7·78	0·003
K (g/d)	1·81	1·44–2·26	2·39	1·72–2·72	1·78	1·43–2·17	<0·001

* Mann–Whitney *U* test.

Multiple logistic regression analysis was conducted to explore the factors related to dietary under-reporters (Table 4). Based on the results of the univariate analyses, the following variables were included in the multiple logistic regression model: pre-pregnancy BMI, weight change during pregnancy, management of gestational weight gain with the aim to return to pre-pregnancy weight soon after childbirth, regularly skipping breakfast and the GSES score. The characteristics independently associated with dietary under-reporters during pregnancy were lower pre-pregnancy BMI (adjusted OR (AOR) = 0·81; *P* = 0·041), lower gestational weight gain (AOR = 0·82; *P* = 0·024), and management of gestational weight gain with the aim to return to their pre-pregnancy weight soon after childbirth (AOR = 2·99; *P* = 0·009). Regularly skipping breakfast and the GSES score were not significantly correlated with dietary under-reporters, after controlling for the other variables.

**Table 4.** Factors related to dietary under-reporting (*n* 271)* (Odds ratios and 95 % confidence intervals)

	Crude OR	95 % CI	*P*	Adjusted OR	95 % CI	*P*
Pre-pregnancy BMI (kg/m^2^)	0·87	0·73, 1·03	0·111	0·81	0·67, 0·99	0·041
Weight change during pregnancy† (kg)	0·89	0·77, 1·02	0·089	0·82	0·70, 0·97	0·024
Management of gestational weight gain with the aim to return to pre-pregnancy weight soon after childbirth (yes = 1; no = 0)	2·64	1·21, 5·74	0·015	2·99	1·31, 6·85	0·009
Regularly skipping breakfast‡ (yes = 1; no = 0)	1·96	0·95, 4·02	0·068	1·88	0·89, 3·97	0·097
GSES§ (score)	0·92	0·86, 1·01	0·082	0·96	0·86, 1·06	0·385

GSES, General Self-Efficacy Scale.

* Multiple logistic regression analysis. Each variable in the table was adjusted for all other variables in the table. The dependent variable is under-reporters (yes = 1; no = 0). Under-reporters are participants with both protein and K under-reporting (*n* 35). ‘Pre-pregnancy BMI’, ‘weight change during pregnancy’ and ‘GSES’ are continuous variables. ‘Management of gestational weight gain with the aim to return to pre-pregnancy weight soon after childbirth’ and ‘regularly skipping breakfast’ are categorical variables (yes = 1; no = 0).

† Weight change during pregnancy refers to the difference between weight at the check-up at 19–23 weeks of gestation and the pre-pregnancy weight: ((weight at the check-up at 19–23 weeks of gestation) – (pre-pregnancy weight)).

‡ Regularly skipping breakfast was defined as forgoing breakfast including a staple food, such as rice or bread, two or more times per week.

§ Higher scores of the GSES indicate a higher level of self-efficacy (0–16 points).

## Discussion

In the present study, we examined factors related to under-reporters in a dietary assessment questionnaire among Japanese women during the second trimester of pregnancy. The characteristics associated with under-reporters, who were classified as both protein and K under-reporting, were lower pre-pregnancy BMI, lower gestational weight gain, and management of gestational weight gain with the aim to return to their pre-pregnancy weight soon after childbirth.

We sought to better understand dietary under-reporters by identifying under-reporting using 24-h urinary excretion of protein and K. This method was based on previous studies that showed that under-reporting of protein and K intakes was strongly associated with under-reporting of energy intake^(^^29^^,^^30^^,^^50^^)^. The cut-off points of under-reporting, defined as the bottom 25 % of the reporting accuracy for each nutrient in the present study, were stricter than the theoretical figure of 1·0. Thus, the under-reporters identified in the present study were presumed to have severely under-reported their dietary intake.

Participants with lower pre-pregnancy BMI were more likely to under-report their dietary intake. This result differed from the results of previous studies of non-pregnant women in other countries, which reported that overweight and obese women tend to under-report their dietary intake owing to their desire for weight loss^(^^1^^,^^4^^,^^5^^,^^8^^)^. In contrast, a Japanese study^(^^13^^)^ reported that most dietary under-reporters among young non-pregnant women were relatively lean. Japan has a unique situation in that the rate of underweight women is higher and the rate of overweight and obese women is lower than the rates in other developed countries^(^^51^^)^. The rate of pre-pregnancy underweight women in our study (19 %) was far higher than those in other developed countries (3 and 2 % in the USA and Portugal, respectively)^(^^52^^,^^53^^)^. Furthermore, young Japanese women are likely to desire weight loss and to perform unnecessary weight control, even though their BMI indicates that they are underweight or have normal weight^(^^54^^,^^55^^)^. Such cultural differences in the attitudes towards weight might have contributed to the opposite findings in the present study. Our results suggest that lower pre-pregnancy weight should be considered a risk factor for dietary under-reporting in Japanese dietary surveys.

Dietary under-reporters had significantly lower gestational weight gain at 19–23 weeks of gestation. The lower gestational weight gain might have been partly due to severe nausea and vomiting during pregnancy because women with nausea and vomiting had significantly lower gestational weight gain at 19–23 weeks of gestation. However, if ingested foods were lost by vomiting, the reporting accuracy would be skewed towards over-reporting because the urinary excretion levels of nutrients would be lower than the ingested amounts. Thus, this opposite result is noteworthy. One possible explanation is that women who vomited after eating might not report the dietary intake, even if the foods they ate were partially absorbed. In addition, despite their efforts, participants with nausea and vomiting often feel that they do not have adequate dietary intake for themselves and their babies^(^^56^^)^. Therefore, a contrast between reality and desire might result in differences between true and perceived intakes, specifically in underestimation of the actual quantity consumed. Healthcare professionals should recognise the possible effects of severe nausea or vomiting on under-reporting when they assess self-reported dietary intake during pregnancy.

Moreover, dietary under-reporters were more likely to manage their gestational weight gain with the aim to return to their pre-pregnancy weight soon after childbirth. It is well known that excessive gestational weight gain contributes to postpartum weight retention^(^^57^^)^. Most of our participants had a desire to return to their pre-pregnancy weights, similar to the findings of another study of pregnant Japanese women (82 and 86 %, respectively)^(^^25^^)^. Management of gestational weight gain often leads to dietary restriction. The relationship between dietary restriction and under-reporting has been observed in non-pregnant populations^(^^58^^,^^59^^)^. On the other hand, managing weight gain for the prevention of pregnancy complications and difficult deliveries was not associated with dietary under-reporting in the present study. Pregnancy complications and difficult deliveries may result from either too little or excessive weight gain; however, only excessive weight gain is of concern in order to return to pre-pregnancy weight after childbirth. Thus, management of weight gain with the aim to return to their pre-pregnancy weight soon after childbirth might be an alternative indicator of dietary restriction during pregnancy.

Regularly skipping breakfast and the GSES score were not significantly correlated with dietary under-reporters, although they were included in the multiple regression model based on the results of the univariate analyses. Further research on the relationships between these variables and dietary under-reporting is required. In particular, dietary-specific self-efficacy might directly influence dietary reporting accuracy, although we could not assess this relationship due to the lack of a validated dietary-specific self-efficacy scale for pregnant women.

In contrast to previous studies of non-pregnant women, social desirability and social approval motivation were not associated with dietary under-reporting during pregnancy^(^^19^^,^^60^^)^. The internal subscale of the FHLC was also not associated with the reporting accuracy of dietary intake, although we hypothesised that pregnant women with strong beliefs that their healthy lifestyles positively affect the well-being of their fetuses might misreport dietary intake by choosing to give socially desirable responses. In the prenatal period, however, socially desirable dietary habits vary among individuals, because some consider eating a lot of vegetables to be a socially desirable habit, while others consider smaller energy intake to be a socially desirable habit. Even if social desirability or social approval motivation affected the reporting accuracy, individual differences in the perceived social desirability of healthy diets during pregnancy would have attenuated the effect.

The present study had several limitations. First, the participant characteristics may have been biased because the research was conducted at a single university hospital in an urban area. Further, the mean age of the participants in this study was slightly higher than that of the participants in national reports (34·5 *v.* 31·7 years)^(^^61^^)^. Although the dietary intakes of our participants were equivalent to in similar reports, differences in the demographic characteristics might potentially have affected the dietary intake. Second, our sample size was relatively small. Especially, only thirty-five under-reporters were identified. Thus, the results should be interpreted cautiously. Further large-scale studies are required to confirm the results of the present study. Third, in consideration of feasibility, a single 24-h urine collection was conducted although multiple 24-h urine collections are recommended to alleviate the influence of day-to-day variations^(^^62^^)^. However, the excretion levels of protein and K would probably reflect habitual intake, because they have few changes by the day^(^^48^^,^^63^^)^. Fourth, the present study used the DHQ to collect self-reported dietary data. The DHQ includes 150 selected foods and beverage items. These 150 items were chosen based on foods that Japanese people typically eat. Thus, we consider that the DHQ can estimate the dietary intake in most participants. However, if the participants prefer to eat uncommon foods that are not included in the DHQ, the estimation of dietary intake might be insufficient. Fifth, we set up relative cut-off points of under-reporting. We speculated that the under-reporters identified in our study under-reported dietary intake heavily, because the cut-off point was a strict criterion. However, the present results might be partially affected by the chosen cut-off value. Sixth, the pre-pregnancy weights used in the present study were self-reported. Healthcare professionals always ask pregnant women about their pre-pregnancy weight at the first check-up of pregnancy and describe the values in the medical charts and the maternal and child health handbooks. Accordingly, we consider that the self-reported values (same as in the medical charts and the maternal and child health handbooks) are fairly reliable.

In conclusion, the findings of the present study revealed the following characteristics of dietary under-reporters during pregnancy in Japan: lower pre-pregnancy BMI, lower gestational weight gain, and management of gestational weight gain with the aim to return to pre-pregnancy weight soon after childbirth. Healthcare professionals should consider the effects of pregnancy-specific weight-related variables on dietary under-reporting when assessing the dietary intake of pregnant Japanese women. However, the sample size of the present study was small. Thus, larger studies are required to confirm the factors related to dietary under-reporting during pregnancy.
